# Handgrip strength effectiveness and optimal measurement timing for predicting functional outcomes of a geriatric hip fracture

**DOI:** 10.1038/s41598-022-25177-3

**Published:** 2022-11-29

**Authors:** Jeongae Han, Chul-Ho Kim, Ji Wan Kim

**Affiliations:** 1 University of Ulsan College of Medicine, Seoul, Republic of Korea; 2 Department of Orthopaedic Surgery, Chung-Ang University Hospital, Chung-Ang University College of Medicine, Seoul, Republic of Korea; 3 Department of Orthopaedic Surgery, Asan Medical Center, University of Ulsan College of Medicine, 88 Olympic-ro 43-gil, Songpa-gu, Seoul, Republic of Korea

**Keywords:** Diseases, Rheumatic diseases, Trauma

## Abstract

Handgrip strength (HGS) now draws attention as one of the predictors of outcomes following geriatric hip fracture; however, its effectiveness and the optimal time to assess HGS remain unknown. Herein, we aimed to determine the usefulness of HGS in predicting the outcomes of geriatric hip fracture and to find the most effective time to measure HGS in both the low muscle strength and normal hip fracture groups. The study was performed prospectively for 79 geriatric hip fracture patients. HGS was measured during the admission period and the one-week postoperative period. Walking ability and quality of life were assessed using Koval scores and the European Quality of Life Five Dimension (EQ-5D) scale at the admission period and postoperatively at 3, 6, and 12 months, respectively. The relationship between pre/postoperative HGS and functional outcomes was assessed, and the functional score between the “low muscle strength” and “normal muscle strength” groups was compared. The association between HGS asymmetry and low strength with functional limitations was determined. For the preoperative HGS, the Koval score showed a significant relationship in the postoperative 6-month (r = −0.295, P = 0.008) and 12-month (r = −0.266, P = 0.019) periods; also, the EQ-5D score showed a significant relationship in the postoperative 6-month and 12-month periods (r = 0.344, P < 0.001, and r = 0.386, P = 0.001, respectively). For the postoperative HGS, the Koval score showed a significant relationship in the 6-month (r = −0.432, P < 0.001) and 12-month (r = −0.344, P = 0.002) postoperative periods. Also, the EQ-5D score showed a significant relationship in the 3-month (r = 0.340, P = 0.010), 6-month (r = 0.476, P < 0.001), and 12-month (r = 0.471, P < 0.001) postoperative periods. The incidence of preoperative and postoperative low HGS was 78.5% and 70.9%, respectively. The “low-strength” group had poor Koval scores and EQ-5D at postoperative month 12 and poor functional outcomes earlier in the follow-up (postoperative 6- and 12-month Koval scores and postoperative 3-, 6-, and 12-month EQ-5D), respectively (P = 0.008 and P = 0.003; P = 0.003, P = 0.001, and P = 0.001). The effect of HGS asymmetry and low strength on functional limitations remained undetermined. Both preoperative and postoperative HGS reflected functional outcomes of patients with hip fracture during the 12-month follow-up. Postoperative HGS had a higher prognostic value than preoperative HGS.

## Introduction

Hip fracture is a common but serious injury that increases in incidence with the aging of the population^1^. As the older adult population increases worldwide, hip fracture incidence is expected to increase 1.7-fold from 2016 to 2025, and threefold between 2016 and 2050^2,3^. Hip fracture is a major health problem associated with high mortality, morbidity, and cost^4–6^. An effort to identify prognostic factors for hip fracture has revealed that sarcopenia, age, previous functional status, medical history of dementia, and delirium during admission as predictors of functional recovery^7,8^. Sarcopenia, an important quantifiable factor, is a geriatric syndrome characterized by the loss of muscle mass and function with aging^9^. Hand grip strength (HGS) has been used as an important index of low muscle strength to diagnose sarcopenia^10^, and is promising because it is a simple and reliable prognostic indicator for hip fractures^11^.

Various studies have investigated the relationship between HGS and the functional outcome of geriatric hip fractures; however, there are still a lot of controversial results^12–20^. Although some studies have demonstrated no relationship between HGS and functional outcomes^16,20^, most were retrospective studies with relatively short follow-up periods of less than 6 months. Additionally, a difference in HGS according to the timing of the measurement (preoperative vs. postoperative) has been observed, begging the question of which measurement would hold better predictive value for functional outcomes after hip fracture. Some researchers measured the HGS of patients with hip fractures immediately after hospital admission based on the maintained muscle mass during the acute post-fracture stage^14,15,19,21^. Other studies that used HGS data after surgery to predict hip fracture risk or investigate hip fracture prognosis showed contradictory results^13,17^.

Therefore, this study aimed to clarify the effectiveness of HGS by analyzing its relationship with functional outcomes, such as Koval scores and the European Quality of Life Five Dimension (EQ-5D) scale, in patients with geriatric hip fracture and to demonstrate the optimal timing for HGS measurement as a prognostic indicator for functional outcomes.

## Materials and methods

### Study design and patients

This was a prospective, observational study of patients with hip fracture in a single center (university hospital) from June 2018 to December 2019. The study was approved by our institutional review board, and all patients provided their written informed consent. All of the consecutive patients meeting the following inclusion criteria were enrolled: (i) aged ≥ 60 years; (ii) had undergone operative treatment of proximal femoral fracture, which was defined as a femur neck fracture or intertrochanteric femoral fracture (AO/OTA 31)^22^, and subtrochanteric fracture (fractures extending 5 cm below the lower border of the lesser trochanter)^23^; and (iii) low-energy injury due to falls from heights of 1 m or less^24^. We excluded patients with: (i) pathologic fracture, (ii) inability to walk before fracture, (iii) multiple fractures, such as combined fragility upper extremity fractures, ipsilateral lower extremity fractures, or bilateral hip fractures, (iv) prophylactic fixation, (v) periprosthetic fracture, and (vi) delayed surgery with neglected fracture.

### Treatment details

Patients with femoral neck fracture underwent bipolar hemiarthroplasty or internal fixation with multiple screws, whereas those with intertrochanteric and subtrochanteric fractures underwent intramedullary nailing. All patients underwent the same standardized postoperative rehabilitation program and were encouraged to practice early assisted ambulation. Patients underwent wheelchair ambulation on the first postoperative day, followed by standing exercises and tolerable weight-bearing exercises with a walking aid (walker or crutches) supervised by a physiatrist and a therapist. All patients were generally discharged 5–7 days after admission and were followed up at postoperative week 4, and month 3, 6, and 12.

### Data collection

Patient demographics were collected, including age, sex, height, weight, body mass index (BMI), diagnosis (fracture site), time between admission and surgery, and length of stay.

### Measurement of HGS

HGS was measured according to the 2019 AWGS recommendation^6^, using a Takei digital grip strength dynamometer (Model T.K.K.5401, Takei, Niigata, Japan). Patients performed isometric contraction a total of six times by alternating both hands. The patients were instructed to grip as hard as possible^25^, and the data from the best performance HGS from both hands were used for the analyses. The HGS was measured with 90° elbow flexion in the sitting position on a bed or chair. However, the preoperative test was performed in the supine position due to pain limiting the sitting position. The preoperative HGS was measured at initial ward admission, and postoperative (pre-discharge) HGS was measured at approximately 1 week post-fracture. According to the diagnostic cutoff value of the 2019 Asian Working Group for Sarcopenia (AWGS)^6^, HGS is defined as “low strength” when the weight is less the 26 kg for men and 18 kg for women. Patients who had a HGS ratio of < 0.90 or > 1.10 were considered to have asymmetric HGS, whereas those with a HGS ratio of 0.90–1.10 had HGS symmetry^26^.

### Evaluation of functional outcomes

Functional outcomes were measured by the Koval score to assess walking ability as a patient-reported outcome and the EQ-5D VAS scale, an international method that reflects the quality of life. The Koval score ranges from 1 to 7, indicating physical condition according to the degree of walking^27^. A higher score reflects a poorer walking ability^28^. The Koval score was measured during the admission period (preoperative period: initial ward admission), at 6 and 12 months post-fracture. The EQ-5D scale was evaluated at admission, 3, 6, and 12 months post-fracture. Functional limitation was defined in the analysis by categorizing patients with “functional limitation” when their Koval score was IV, V, VI, and VII or their EQ-5D was less than 0.5^29,30^.

### Statistical analysis

The correlations between HGS and Koval scores and EQ-5D for each period were assessed to investigate the relevance to functional recovery. We evaluated the Spearman’s rank correlation coefficient for the relationship between preoperative and postoperative HGS and functional outcomes. After performing the Kolmogorov–Smirnov normality analysis and confirming non-normal distribution, the Mann–Whitney U test was conducted to assess the differences in functional outcomes between the low-strength and normal groups. Patients were classified into four groups according to their handgrip asymmetry and strength: (1) low strength only, (2) asymmetry only, (3) asymmetry and low strength, and (4) symmetric and normal strength. Logistic regression models were used to determine the associations between the low strength and HGS asymmetry on functional limitations using group ‘symmetric and normal strength’ as a reference. All logit models were adjusted for sex, age, height, weight, BMI, psoas muscle volume, and preoperative diagnosis using multivariate analysis. All the statistical analyses were performed using IBM SPSS Statistics for Windows, version 25.0 (IBM Corp., Armonk, NY, USA), and significance was accepted for P-values of < 0.05.

### Ethical approval

All procedures involving human participants were performed in accordance with the ethical standards of the institutional committee. This study was approved by the Institutional Review Board of Asan Medical Center (protocol no. 2018-0932).

### Informed consent

Informed consent was obtained from all participants.

## Results

**Figure 1:**
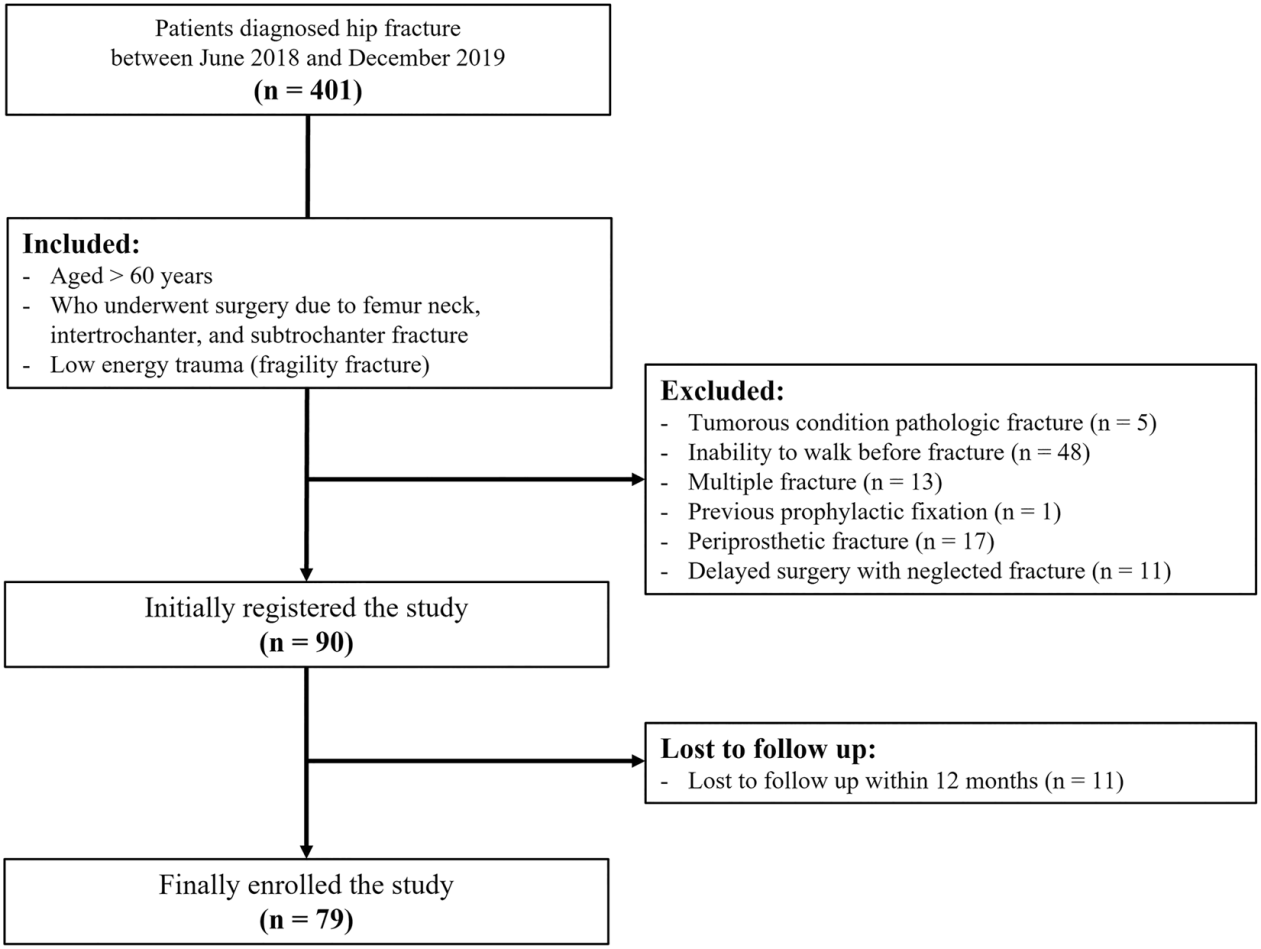
Flow diagram of patient selection.

The details of patient selection and the number of patients included in the following steps are shown in Fig. 1. A total of 79 patients with a mean age of 76.1 ± 7.9 years (range, 60–96 years) participated in the study, 75.1% of whom were women. The mean BMI was 23.0 ± 3.8 kg/m^2^ (range, 15.2–35.7 kg/m^2^). There were 38 cases (48.1%) of femur neck fracture, 29 (36.7%) of intertrochanteric fracture, and 12 cases (15.2%) of subtrochanteric fracture. The average time interval from admission to surgery was 1.8 ± 2.5 days (range, 0.2–16.8), and the length of stay was 9.3 ± 7.0 days (range, 4–56). After preoperative HGS assessment, 62 patients were assigned to the low-strength group, while postoperative HGS analysis assigned a further 56 cases (70.9%). Two patients died during follow-up at postoperative months 6 and 12. Their data were collected up to 6 months after fracture (Table 1).

**Table 1:** Baseline characteristics.

Variables	All
Total number (%)^a^	79 (100)
Age, years	76.1 ± 7.9
**Sex**	
Male, n (%)	19 (24.1)
Female, n (%)	60 (75.9)
Height, cm	158.3 ± 7.9
Weight, kg	57.8 ± 10.3
BMI, kg/m^2^	23.0 ± 3.8
Preoperative HGS (total)	15.5 ± 7.6
Low strength, n (%)	62 (78.5)
Normal, n (%)	17 (21.5)
Male	22.2 ± 11.0
Female	13.4 ± 4.8
Postoperative HGS (total)	16.8 ± 8.2
Low strength, n (%)	56 (70.9)
Normal, n (%)	23 (29.1)
Male	24.7 ± 9.8
Female	14.3 ± 5.7
Psoas muscle volume (total)	324.0 ± 430.3
Psoas muscle cross-sectional area—L3 (total)	1314.1 ± 499.5
Psoas muscle cross sectional area—L4 (total)	1605.4 ± 54.0
Preoperative VAS score	2.8 ± 1.3
Postoperative VAS score	1.6 ± 1.0
Preoperative EQ-5D (total)	
Preoperative Koval score (total)	
**Preoperative diagnosis**	
Femur neck Fx (%)	38(48.1)
Intertrochanteric Fx(%)	29(36.7)
Subtrochanteric Fx (%)	12(15.2)

*BMI* body mass index, *Fx* fracture, *VAS* visual analog scale, *SD* standard deviation.

^a^Calculated the result rounded up to the first digit after the decimal point.

The relationships between pre/postoperative HGS and Koval scores are shown in Table 2. Both HGS was negatively correlated with Koval scores at postoperative months 6 (−0.295 in preoperative and −0.432 in postoperative) and 12 (−0.266 in preoperative and −0.344 in postoperative), with a stronger correlation and higher correlation coefficient observed at the postoperative HGS assessment (P = 0.001). The relationships between pre/postoperative HGS and EQ-5D scores are shown in Table 3. Patients with higher preoperative HGS had higher EQ-5D scores at postoperative months 6 and 12 (P = 0.008 and P = 0.003), whereas higher postoperative HGS was correlated with higher EQ-5D scores at postoperative months 3, 6, and 12 (P = 0.003, P = 0.001, and P = 0.001).

**Table 2:** Spearman rank correlation coefficient between HGS and Koval score.

Variables	Koval score		
	Preoperative period	6 months postoperatively	12 months postoperatively
Preoperative HGS	−0.184 (P = 0.104)	−0.295 (P = 0.008)	−0.266 (P = 0.019)
Postoperative HGS	−0.192 (P = 0.090)	−0.432 (P < 0.001)	−0.344 (P = 0.002)

*HGS* handgrip strength.

**Table 3:** Spearman rank correlation coefficient between HGS and EQ-5D score.

Variable	EQ-5D score			
	Preoperative period	3 months postoperatively	6 months postoperatively	12 months postoperatively
Preoperative HGS	−0.019 (P = 0.900)	0.240 (P = 0.072)	0.344 (P < 0.001)	0.386 (P = 0.001)
Postoperative HGS	−0.320 (P = 0.832)	0.340 (P = 0.010)	0.476 (P < 0.001)	0.471 (P < 0.001)

*EQ-5D* European Quality of Life Five Dimension, *HGS* handgrip strength.

We compared the functional outcomes between the low and normal strength groups (Tables 4 and 5). The low-strength group had higher Koval scores and lower EQ-5Ds scores at postoperative month 12 (Table 4). When compared based on postoperative HGS, the low-strength group had higher Koval scores at postoperative months 6 and 12. The EQ-5D score was significantly lower in the low-strength group at postoperative months 3, 6, and 12 (Table 5).

**Table 4:** Comparison of functional outcomes between the “low strength” group and “normal” group using preoperative HGS (the values are shown as mean ± SD).

Variables	Preoperative HGS (the values are shown as means ± SD)		P-value*
	Low-strength group (N = 62)	Normal group (N = 17)	
Koval score preoperative period	1.92 ± 1.74	1.29 ± 0.85	0.108
6 months postoperatively	2.56 ± 1.88	1.53 ± 0.72	0.052
12 months postoperatively	1.92 ± 1.20	1.18 ± 0.53	0.008
**EQ-5D score**			
PreOp	0.04 ± 0.18	0.07 ± 0.24	0.760
3 months postoperatively	0.65 ± 0.20	0.72 ± 0.13	0.234
6 months postoperatively	0.71 ± 0.23	0.82 ± 0.12	0.165
12 months postoperatively	0.77 ± 0.22	0.89 ± 0.18	0.024

*EQ-5D* European Quality of Life Five Dimension, *HGS* handgrip strength.

*Mann–Whitney U test.

**Table 5:** Comparison of functional outcomes between the “low strength” group and “normal” group using postoperative HGS.

Variables	Postoperative HGS (the values are shown as means ± SD)		
	Low-strength group (N = 56)	Normal group (N = 23)	P-value*
Koval score preoperative period	1.93 ± 1.68	1.43 ± 1.38	0.077
6 months postoperatively	2.70 ± 1.93	1.48 ± 0.67	0.008
12 months postoperatively	2.00 ± 1.25	1.17 ± 0.39	0.003
**EQ-5D score**			
PreOp	0.04 ± 0.19	0.05 ± 0.19	0.552
3 months postoperatively	0.62 ± 0.20	0.78 ± 0.08	0.003
6 months postoperatively	0.69 ± 0.23	0.86 ± 0.11	0.001
12 months postoperatively	0.74 ± 0.22	0.93 ± 0.12	< 0.001

*EQ-5D* European Quality of Life Five Dimension, *HGS* handgrip strength.

*Mann–Whitney U test.

The effect of low strength or weakness on functional limitations represented by the Koval score was not identified (Table 6). Patients with low strength, HGS asymmetry, or both showed no significant difference in functional limitations compared with patients with HGS symmetry and normal strength.

**Table 6:** The association between “low strength” and “HGS asymmetry” and functional limitations.

Groups	Odds ratio	95% confidence interval	P-value*
Low strength only	6.00	0.290, 124.1	0.246
HGS asymmetry only	2.160	0.207, 22.48	0.519
HGS asymmetry and low strength	3.913	0.419, 36.532	0.231

*Logistic regression.

## Discussion

Our study demonstrated that both preoperative and postoperative HGS reflected postoperative walking ability and quality of life; however, postoperative HGS had a higher prognostic value with functional outcomes than preoperative HGS. The absolute values of the correlation coefficients of postoperative HGS related with Koval scores at postoperative months 6 and 12 and the correlation coefficients of postoperative HGS related with EQ-5D scores at postoperative months 6 and 12 were higher than those at preoperative HGS analysis. In addition, the rate of low strength was significantly lower at postoperative HGS.

This study reconfirmed the importance of HGS as a prognostic factor of functional outcome in patients with hip fracture. The results are consistent with those from previous studies, confirming the prognostic role of HGS after hip fracture^12–15,17–19^. However, there was no consensus on the timing of HGS measurement, and measurements were taken at inconsistent times throughout these studies. Several studies evaluated preoperative HGS. Monaco et al. demonstrated that grip strength better predicted short-term functional outcomes in women than appendicular lean mass assessed by dual-energy X-ray absorptiometry^12^. Álvarez et al. concluded that HGS measured at admission for hip fracture was directly related to functional recovery in older patients^19^. Selakovic et al. showed that HGS measured preoperatively was associated with Barthel index scores at months 3 and 6 post-fracture^15^. Furthermore, Wehren et al. found a moderate correlation between grip strength and functional outcomes^18^. Savino et al. also concluded that high HGS at admission was related to a higher probability of independent walking recovery within 1 year of surgery^14^. Other studies revealed the relationship between postoperative HGS and functional outcomes. Monaco et al. assessed HGS before rehabilitation and observed the prognostic ability of functional outcome of inpatient rehabilitation and at 6 months follow-up in women with hip fractures^13^. Beloosesky et al. demonstrated that HGS measured 1 week after surgery was associated with the recovery of walking ability at postoperative month 6^17^.

Only two studies found no relationship between grip strength and functional outcome. Steihaug et al. demonstrated that postoperative HGS was not significantly associated with the preoperative Barthel index^16^. Their study differed from the current study in the relationship between HGS and ‘pre-injury’ functional status was demonstrated, rather than functional outcome after fracture. Gonzalez-Montalvo et al. also failed to show the association between sarcopenia and short-term functional outcome in patients with hip fracture^20^. Limited comparisons can be drawn between this study and our present study because they did not assess the direct relationship between HGS and long-term functional outcome, including walking ability and activity of daily living, and their functional outcome was measured only at discharge at an average of 10.1 days, without further follow-up.

With respect to the time of HGS assessement, the current study revealed that postoperative HGS reflected prognosis better than preoperative HGS. The correlation coefficient of postoperative HGS with functional outcome was higher than that of preoperative HGS. In addition, postoperative HGS showed a moderate correlation with postoperative 6-month walking ability and postoperative 6- and 12-month EQ-5D. In contrast, preoperative HGS showed a weak correlation with postoperative 6- and 12-month walking ability and EQ-5D score. Although the low strength group based on preoperative HGS showed a lower walking ability and quality of life only at postoperative month 12, the same group based on postoperative HGS had lower walking abilities at postoperative months 6 and 12 and lower EQ-5D scores at postoperative months 3, 6, and 12. Therefore, we believe that postoperative HGS better reflects functional outcomes after hip fracture and is useful in predicting postoperative 6- and 12-month walking ability and quality of life.

The higher prognostic value of postoperative HGS may be attributed to the following reasons. Muscle mass is maintained during the first 10 days after fracture and begins to decrease thereafter^31,32^. The time difference between pre- and postoperative HGS was approximately 6 days (< 10 days); thus, the difference in HGS may not have resulted from the change in muscle mass between the measurements. First, preoperative HGS was highly influenced by pain, which would have restricted the maximum force exercised by the patients in the preoperative setting. Moreover, preoperative HGS was measured in the supine position due to hip pain, which is not the standard measurement method advocated by the American Society of Hand Therapists^33^. Teraoka^34^ reported that HGS in the supine position was weaker than that in the standing or sitting positions, owing to the influence of gravity. Therefore, we believe that the preoperative HGS could have been underestimated.

Additionally, postoperative 1-year mortality rates in geriatric hip fractures are 16% in South Korea^35^. One-year mortality in the current study was 2.5%, which is much lower than that of studies in the literature. We suggest that two factors might have attributed to lower mortality rates in our study. This study was a prospective cohort study, excluding patients unable to walk before fracture or delayed fracture; agreeing participants were therefore relatively healthy and active. Second, the current study was performed in a well-organized center for geriatric patients, with all surgeries performed as soon as possible by an experienced orthopedic surgeon. Integrated co-management for elderly patients and experience of the surgeon with hip fracture would reduce hospital mortality.

We also examined the associations between HGS asymmetry and weakness in functional limitations. Our analysis revealed the effect of low strength or weakness on the functional relationship, although none were statistically significant. Our study has therefore been unable to determine whether asymmetry or low strength affects functional limitation before surgery. The reliability of the study may also have been impacted due to the large difference in the number of patients in each group; (1) low strength only (n = 28), (2) asymmetry only (n = 4), (3) asymmetry and low strength (n = 28), and (4) symmetric and normal strength. (n = 19). A follow-up study would include an increase in the number of patients and intentions to study the effect of asymmetry and low strength on functional limitations before and after fracture.

There are several limitations to this study. First, this was a single-center study in a tertiary hospital, with all admitted patients of Asian descent. External validity is therefore required to support the global application of our findings. Second, two patients were lost to follow-up at 12 months, which may have affected the statistical analysis. Additionally, admission to surgery and length of stay intervals varied substantially. Patients with long hospital stays (especially 56 days) were included because of their poor condition and rehabilitation requirements. We confirmed that this did not affect the results, but the reliability may have been impacted. In addition, the functional outcomes in our study differed from those in previous studies. The following modalities were used in previous studies: Barthel index^12,13,15,16,19,20^; Functional Ambulation Category^20^; Functional Independence Measure^17^; Activities of Daily Living^18^; and Instrumental Activities of Daily Living^14^. Further studies may be needed to determine the most effective scoring system for measuring functional outcome in patients with hip fracture, or to determine whether the type of scoring system affects the relationship with HGS.

This study also had several strengths. First, we provided postoperative 1-year functional outcomes. Among previous studies, few had follow-up periods of 12 months^14,18^, of those that did, most were less than 6 months^12,13,15–17,19^. Second, previous studies were limited to one-time measurements of HGS^12,13,16,17,19^; however, the current study evaluated preoperative and postoperative HGS and determined the most meaningful measurement. To the best of our knowledge, this is the first study to compare the prognostic value of preoperative and postoperative HGS. In addition, we increased the validity of our study by using two indicators, walking ability and quality of life, as the functional outcomes. Our study has clinical importance in that by evaluating the value of HGS as a prognostic factor and providing an appropriate timing of HGS measurement, its value is maximized. In clinical practice, postoperative HGS measurement would be more valuable and convenient for patients and physicians when the patient is stable and in a comfortable environment without pain.

## Conclusion

In this study, we confirmed that preoperative and postoperative HGS are correlated with postoperative walking ability and quality of life after hip fracture. The postoperative HGS had a higher prognostic value for functional outcomes in patients with hip fractures than preoperative HGS. Thus, postoperative HGS is recommended to better predict the functional status of patients with hip fractures in a comfortable postoperative setting.

## Data Availability

We did not add supplement data and materials in submission but they are available upon request to the corresponding author if required.
